# A customised method for estimating light transmission efficiency of the horizontal light pipe via a temporal parameter with an example application using laser-cut panels as a collector

**DOI:** 10.1016/j.mex.2021.101339

**Published:** 2021-04-24

**Authors:** Biljana Obradovic, Barbara Matusiak

**Affiliations:** a Norwegian University of Science and Technology (NTNU), Trondheim, Norway; b Norconsult AS, Sandvika, Norway

**Keywords:** Horizontal light pipes (HLP), Photometry method, Transmission efficiency, Light deflection panels (LCP)

## Abstract

The purpose of the study presented in this paper was to develop a customized method for estimating the transmission efficiency of a horizontal light pipe (HLP). The proposal is based on the established method and customized to encompass real incident daylight that horizontal light pipes could collect. Natural incident light is introduced using both direct and diffuse light sources through the temporal parameter of their altitude and azimuth. The temporal parameter is presented as a matrix model consisting of solar altitude and azimuth used in measurement and analysis. Because each location on Earth is characterized by a unique solar altitude and azimuth during the year, the proposed method establishes the measuring template, based on a specific location on Earth and applicable for the HLP. The methodology is supplemented with a theoretical model of an indoor space equipped with an HLP that can be used to analyse data and validate necessary threshold illuminance under real physical conditions. An example of an application of the customized method is demonstrated using a case of light collectors for the HLP light-deflecting panels, the laser-cut panel (LCP).

• *A method for predicting the transmission efficiency of the HLP for any location and any orientation on a building's façade.*
• *Estimation of transmission efficiency for the HLP implementing a temporal parameter through solar altitude and azimuth.*
• *Resulting data for transmission efficiency for the HLP using a template that is easily applicable for decision-making during a specific period of the year, season or the entire year.*

## Graphical abstract

**Figure:**
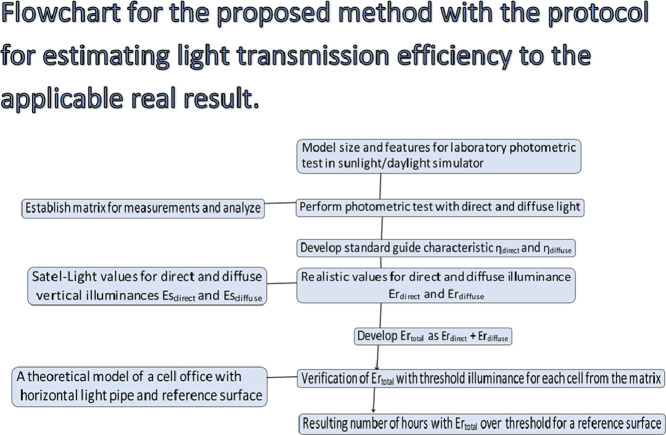

## Nomenclature

*η*: light transmission efficiency
*η_Light Guide_*: light transmission efficiency for a light guide (light pipe)
*η_Collector_*: light transmission efficiency for a light collector
*η_Diffuser_*: light transmission efficiency for a light diffuser
*Φ_o_*: light flux at the light pipe's entrance, lumen
*Φ_1_*: light flux at the light pipe's exit, lumen
*Φ_2_*: light flux at the light pipe's exit, pipe has collector or diffuser, lumen
*E*_direct_(ZERO), *E*_diffuse_(ZERO): direct or diffuse measured illuminance at the tube's entrance, lux
*E*_direct_(BaseCase), *E*_diffuse_(BaseCase): direct or diffuse measured illuminance at the tube's exit, lux
*η_direct_, η_diffuse_*: transmission efficiency for direct or diffuse light
*Es_direct_, Es_diffuse_*: direct or diffuse illuminance on a vertical south façade developed from the Satel-Light, lux
*Er*_direct_, *Er*_diffuse_: direct or diffuse real expected illuminance at the tube's exit, lux
*Er*_total_: total real expected illuminance at the tube's exit, lux
Al, Az: solar altitude and azimuth, degrees

Specifications table

**Table:** 

Subject Area:	Engineering
More specific subject area:	*Improving daylighting techniques and technology to increase the energy efficiency of buildings and enhance daylighting in indoor space for health and wellbeing*
Method name:	*Photometry method for horizontal light pipes and components, addressing real daylight condition;*
Name and reference of original method:	“Method of photometry of Tubular Daylight Guidance systems and components” in CIE Technical report 173:2012 Tubular daylight guidance systems
Resource availability:	*Daylight Laboratory at NTNU, Department of Architecture and Technology; Satel-Light database at*http://www.satel-light.com/

### Background

A review of the literature on daylight transport systems (DTS) revealed that light pipes could reduce the energy used for electrical lighting in commercial buildings at high latitudes by up to 30% [3,^6^,^9^,15]. In particular, horizontal daylight tubes mounted in the ceiling plenum have been promoted as a successful solution for a deeper daylighting supplement in multi-storey commercial buildings in locations far north or south [17]. Horizontally-mounted daylight tubes, unlike vertical light tubes, have a limited possibility to receive light from the zenithal part of the sky and will primarily base its light transmission efficiency on direct light from the sun. Locations far north or south are characterized by low solar altitude. Because the sun's position varies throughout the day, from morning in the east to evening in the west, the efficacy of the horizontal light tube varies in its ability to reach the maximum when light rays are aligned to the tube's longitudinal axis (central axis of light transmission). For buildings at high northern latitudes, the most successful orientation for horizontal tubes is to the south; this orientation coincides with the sun's longest exposure during the year (in winter the sunlight is accessible only from the south).

Light pipes are tubular hollow elements that can be layered with mirror folium, aluminium or silver. The tubes have a reflectivity (R) up to 99%. Light rays with an axillary incident angle, where light rays go straight along the pipe and do not inter-reflect with the pipe's internal surface, contribute the most to the efficiency of light transmission. Any deviation from the axillary incidence angle increases the number of interreflections, and the total light transmittance of the pipe decreases because the inner surface is not 100% reflective. That effect also means that light rays coming from the side, as with morning and evening sun rays for a south-oriented pipe, will intersect the tube entrance at sharp angles and produce numerous interreflections.

The method for estimating horizontal light pipe's efficiency has not been the focus of research in the field of daylighting. Several attempts to find an appropriate and precise method for calculation of light pipe efficiency have been made. However, all of the studies concluded with methods that considered static situations, either a large diffuse light source or a single light incident ray [23], [24], [25]. The Commission Internationale de l'E´clairage's (CIE) Technical report “Tubular Daylight Guidance Systems’’ [2], includes methods to estimate light transmission efficiency only for vertical light pipes. As discussed, the most incident light to the vertical light pipes comes at the zenith. Consequently, the method assumed incident light from a large light source and relies on a specific minimum luminance of the sky [2]. In this case, it was the CIE standard overcast sky with a luminance ratio of 3:1 from zenith to the horizon [8]. This ratio means there is much less diffuse light entering the horizontal pipe than the vertical pipe. This reduced light is the reason why the overall efficiency of the horizontal light pipes, compared to vertical pipes, is low. A result that relied just on an overcast sky induced a lack of interest in the horizontal position for the further research.

Nevertheless, the efficiency of horizontal light pipes may depend more on direct sunlight than on diffuse light from the sky. This is because the entrance to the tube can be directly exposed to low sunlight and result in much higher illuminance than any diffuse skylight can achieve. This higher illuminance aligned with the HLP is the case in the high latitudes of the northern or southern hemispheres.

The incident angle of the incoming light is defined by the sun's azimuth and altitude angles (Al and Az), which can be used as variables describing the temporal parameter. While the original method for estimating transmission did not consider time, the customized method is based on the time of day and the time of year.

The most common way to make a calculation for a period of time was using a simulation in which the period was the critical parameter, and the results showed the “temporal behaviour’’ of a “tested system.’’ In the last three decades, there have been few studies of horizontal light pipes. Some of them were published under the title “simulations’’. They only considered sunlight at a specific altitude/azimuth. No climate-based simulations that encompass the entire year or a specific period during the year have been conducted. The studies were conducted using either Radiance or Trace pro as the light calculation engine, and the model laser-cut panels (LCP) and horizontal light pipes (HLP) previously developed in either CAD [9], Solidworks [20] or mathematical algorithms [7,10]. They resulted in data for a single day or a single sun position. The result was also reflected in the potential for improved daylighting and possible energy savings. However, none of the studies included calculations for an entire year. The likely reason for this was that such a simulation is time-consuming due to the huge number of light rays. Some latest studies addressing LCP and HLP considered laboratory scale model or model “in situ’’ for daylight estimation.

This paper aimed to fill the gap in the development of methodology for estimation of transmission efficacy of horizontal light pipes, and it is written based on a recently performed research study by the same authors [18]. The methodology proposed here can be used to estimate the efficiency of HLP with any entrance orientation. The same method can be used if the research aims to evaluate the efficiency of any custom-made collector that is sensitive to the incident sun angle, such as the LCP, Fresnel lens or anidolic collector. In addition, it shows how the obtained result should be used further to develop realistic data, that would be possible to expect in a repeatable full-scale study. The model for the resulting data can be easily applied to a period or the entire year.

**Fig. 1:**
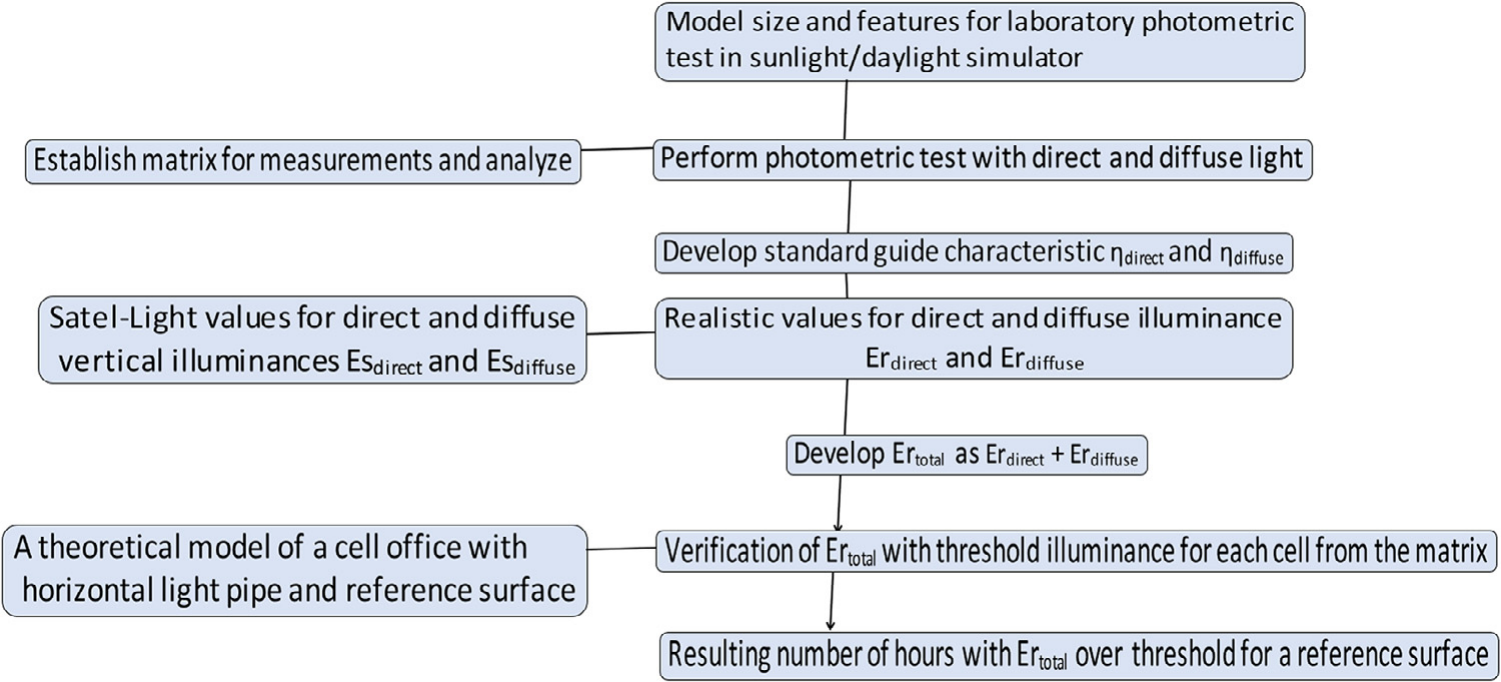
Flowchart for the proposed method with the protocol for estimating light transmission efficiency to the applicable real result.

The original CIE method is summarized in Section *"The existing method for calculating the light transmission efficiency of light pipes"*, while the customized approach is presented in Section *"A customized method for calculating the light transmission efficacy of horizontal light pipes"*. Since the result of such parametric laboratory study is limited to raw data on light transmission efficiency for direct as well as diffuse light conditions, it was necessary to connect them to the realistic daylight values. For this purpose, the information on diffuse and direct illuminance values from the Satel-Light database was used. The original study used Oslo, Norway, as a test location. Section *"Satel-Light database and real daylight accessibility"*presents the developed of the Satel Light data based on that location. Development of the real data is presented in Section *"Development of performance indices using Satel-Light database"*. Section *"The application of real data via the theoretical office model"*describes the application of the real data via a theoretic model of an office space. Section *"Example of an application: an estimate of light transmission efficiency for light deflecting panels as collectors for a horizontal light pipe."*presents an example of a customized method for light transmission efficiency of the LCP as a light collector for the HLP. The protocol presented in this paper was developed through the original research study intended to demonstrate improvements to daylight autonomy using HLP and LCP for buildings in high latitudes [18]. The protocol is described in a flowchart presented in Fig. 1.

### The existing method for calculating the light transmission efficiency of light pipes

**Fig. 2:**
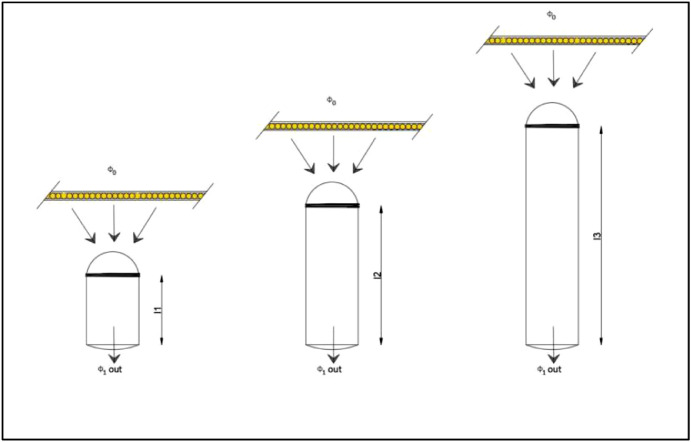
Light guide efficiency, adopted from [2], permission granted from CIE.

**Fig. 3:**
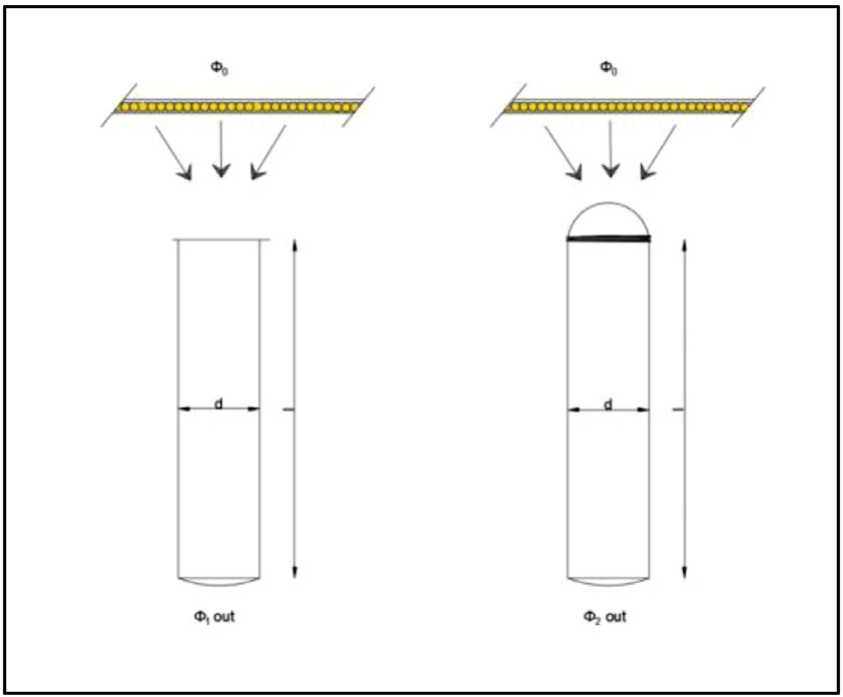
Collector efficiency, adopted from [2], permission granted from CIE.

**Fig. 4:**
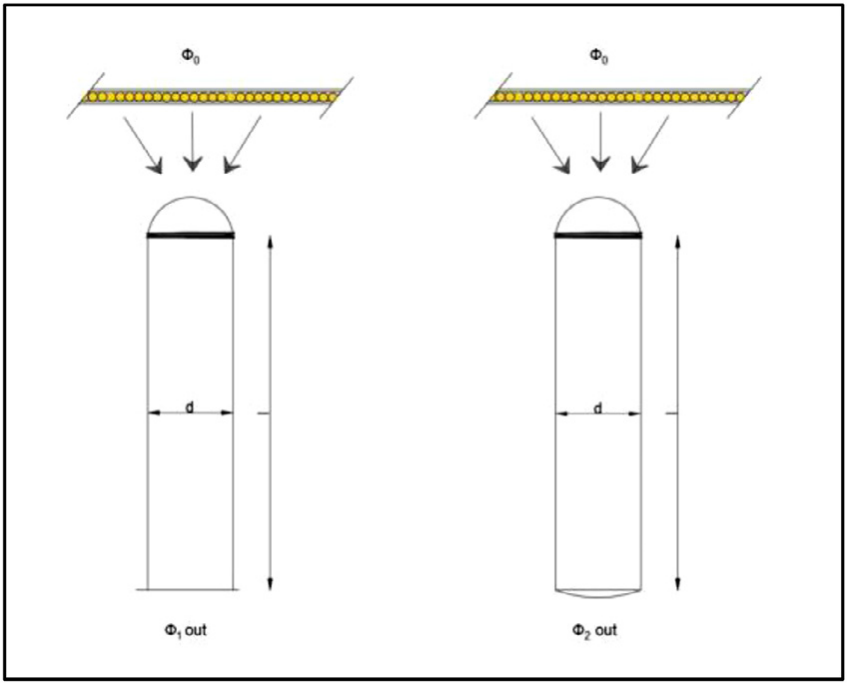
Output device (diffuser) efficiency, adopted from [2], permission granted from CIE.

The CIE 173:2012 technical report: *Tubular Daylight Guidance Systems* presented an approach to determine light transmittance efficiency (η) for vertical light pipes [2]. The photometric estimate can be made using a scale model of the light pipe, accompanied by a collector or diffuser, if desirable. The estimation method is described as “a ratio of luminous flux at the tube's exit and luminous flux at the tube's entrance,’’ (Eq. (1)) in which illumination is provided by “the diffuse light source placed near the tube's input window’’ (Fig. 2). The Φ_o_ represents the input illuminance, and Φ_1out_ represents exit illuminance. The light source is taken as a diffuse illuminance, coming from the zenithal sky (CIE overcast sky). The measuring protocol starts with the reference measurement and continues with the test measurement. The parameter that is evaluated is variated; in the case of tube length, the variable parameter is the length of the tube (Fig. 2). In the case of the collector or diffusor efficiency, the reference measurements are made with no collector or diffusor, while the test measurement has the collector or diffusor (Figs. 3 and 4). The transmission efficiency (*η*) for a specific pipe length is the ratio of outgoing illuminance and input illuminance for all assessed lengths (Eq. (1)). For the transmission efficiency (*η*_Collector_, *η*_Diffuser_) of a light pipe equipped with collector or diffuser, the ratio is between the outgoing illuminance with the collector (diffuser) and outgoing illuminance without the collector (Eqs. (2) and (3)).

(1) $$\eta_{Light\;Guide}=\frac{\phi_{1}out}{\phi_{0}}\times \frac{1}{\eta_{Collector}}$$

(2) $$\eta_{Collector}=\frac{\phi_{2}\;out}{\phi_{1\;}out}$$

(3) $$\eta_{Diffuser}=\frac{\phi_{2}\;out}{\phi_{1\;}out}$$

### A customized method for calculating the light transmission efficacy of horizontal light pipes

Purpose of developing this customized method is to broaden the application of existing method on a horizontal light pipe, and to encompass all daylight conditions. The customized method is a laboratory measurement in Daylight simulator, where, two light sources are used to simulate the sun, and sky, separately. The efficiency of a horizontally-mounted light pipe is, as previously explained, in much higher extend influenced by the direct light coming from the sun than by diffuse light. The relation to the diffuse incident light is static and occurs with overcast skies or with diffuse (exterior and atmosphere reflected) light rays originating from direct sunlight. The light conditions that determine the light transmission efficiency of the HLP are variable; therefore, it is essential to verify efficacy using the full palette of light conditions. Variable light conditions can also be established using a temporal parameter because both direct and diffuse light conditions change with the time of the day and time of year. The proposed customized method considers lighting conditions using a temporally-related altitude-azimuth matrix. The method for developing the altitude-azimuth matrix is explained in Section *"Development of temporal template using a matrix of altitude and azimuth"*. The demands of the scale model study in the sunlight/daylight simulator facilities are presented in Section *"Scale model and laboratory facilities for the parametric measurements"*. The protocol for the photometric measurement and methodology for the development of standard transmission efficiency for an HLP is described in Section *"Laboratory measurements and calculation of the light transmission efficiency (η)"*.

#### Development of temporal template using a matrix of altitude and azimuth

**Fig. 5:**
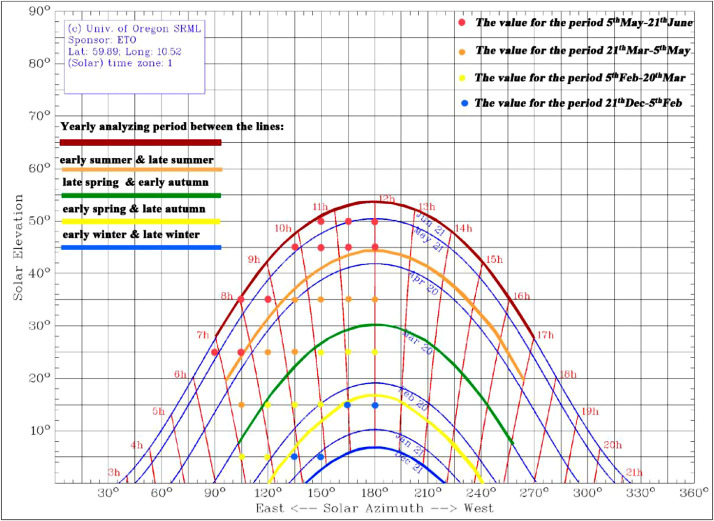
Solar chart for Oslo, Norway (59°53′ N, 10°31′ E) with typical periods used in the analysis and a testing position corresponding to the testing matrix [21]. Test points in colour represent typical analysis periods during the year: red-summer, orange-late spring or early autumn, yellow-early spring or late spring and blue-winter.

Photometric measurements must encompass a temporal parameter that is in direct relation to the sun's altitude and azimuth; they are used to establish the matrix also used as a template in photometric measurements. The protocol is based on an HLP oriented to the south; however, the protocol applies to any light pipe orientation (e.g., east and west or an orientation to the north for the southern hemisphere). Because the matrix points represent testing positions, they must be easy to retrieve under laboratory conditions. As shown in Fig. 5, the testing position is chosen by developing analysis periods based on typical days in the year. For the summer and winter, it is taken the start/end date. The spring and autumn periods have similar solar altitudes and are divided into two periods, early spring corresponding to late autumn, and late spring corresponding to early autumn. This division is done to increase precision because the variations in altitude through spring/autumn are larger than summer/winter. Fig. 5 shows the solar chart for a specific location (Oslo, Norway) from the original study [18] and a matrix of points, each representing 5/10° of the sun's altitude and 15° of the sun's azimuth. The matrix of the solar altitude and azimuth angles was developed by dividing the field of solar incident angles into as many planes as necessary to establish enough points of time. The outermost azimuth angles, 90° and 270° were based on the vertical cut-off of the south façade and the user-occupancy hours.

Due to the complexity and the size of the model, when the method was first demonstrated, altitude 55°, which corresponded best to the highest altitude in Oslo (53°), could not be applied. Instead, the measurements were made at 50° (see Table 1). Test points in colour represent the typical analysis periods of the year: red-summer, orange-late spring or early autumn, yellow-early spring or late autumn and blue-winter. In Fig. 5, the depicted periods, with their characteristic altitude and azimuth, occur two times per year. It is, therefore, easy to develop data for just one period, summer or winter, by multiplying the period by two. The spring (or autumn) period was created by summing data for early and late spring. The total yearly result is developed by multiplying each typical period by the number of days in that period. There are four distinct periods in the year, each period being ¼ of the 365 days or 91.25 days. Each cell in the resulting template contains an illuminance value on the pipe exits (see Section *"Development of performance indices using Satel-Light database"*). The original study, Obradovic and Matusiak [18], proposed a theoretical model for the analysis of that value (see Section *"The application of real data via the theoretical office model"*).

**Table 1:** Testing matrix for the parametric laboratory study referencing the solar chart for a south facade in Oslo, Norway. Test points in color represent typical analysis period of the year: red-summer, orange-late spring or early autumn, yellow-early spring or late autumn and blue-winter [18].

#### Scale model and laboratory facilities for the parametric measurements

A scale model of the horizontal tube to be used for photometric measurements was constructed. The original study was conducted using a model HLP with an aspect ratio (length-to-diameter) of *p* = 1200/150 mm, 8. It represented a 1:2 scale model as the most suitable light pipe module for office buildings. The dimensions of the model were considered in relation to the dimensions of the light simulator in which the photometric analysis was performed. The original study used the Daylight Laboratory at NTNU, Department of Architecture and Technology. The static sunlight source was used for the direct light study and a mirror box with a luminous ceiling was used for the diffuse light study. The distance between the light source and the model (in this case, the entrance of the horizontal light pipe) was the critical parameter because of the potential for a serious parallax error.

The parallax issue was explained as incorrect incident light (luminance pattern) from a light source because of the size of the sunlight/daylight simulator compared to the scale model. In reality, any light source, either the sun or a sky dome, is distant and large. By contrast, any building (or a light pipe) is exceedingly small. This difference means that any opening/window in the actual building would receive equal light in intensity and direction. However, in the sunlight/daylight simulators, light rays from the sun are not perfectly parallel or of equal intensity at all openings or across one large opening in the model. Under laboratory conditions, one sunlight simulator will never be large enough to minimise the parallax issue. It is also possible to account for a dimension of a scale model that cannot be small enough to be compared with real conditions. On the other hand, the scale model must be large enough to retain the details essential for light simulation and photometric measurements [1]. If the model's entrance to the pipe is too big, the entrance surface will not receive an equal intensity of ‘’parallel’’ sunlight rays from a sunlight simulator. Therefore, it is important to ensure that the tube entrance will be located in the parallax-bounded volume with the highest predicted accuracy (+/−10%) [12].

**Fig. 6:**
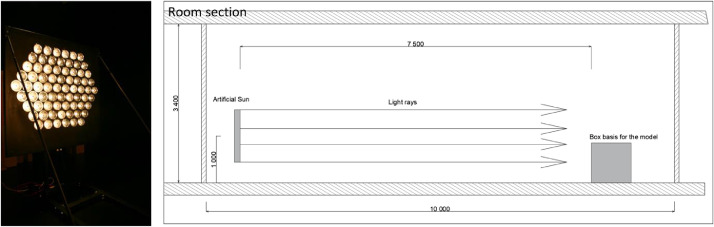
Artificial sun setup in the Daylight laboratory at NTNU, Faculty of Architecture and Design, photo of the artificial sun to the left and section of the room to the right [18].

The original study was conducted in the Daylight Laboratory at NTNU. The direct sunlight facility there is in the form of a static artificial sun. The artificial sun is composed of 70 halogen lamps with parabolic reflectors (50 W) fixed to a vertical metal plate and arranged in a hexagonal pattern. The artificial sun provides near-parallel light beams with a dispersion angle of 3° It was situated in a corridor-like room, enabling sufficient distance from the sun to the model (Fig. 6). The walls, ceiling, and floor were painted matte black to minimize interreflections in the room and minimize scattering light on the model. The model (light pipe) was positioned 7.5 m from the artificial sun, ensuring even illumination. The uniformity of the light from the artificial sun on the tube's entrance, measured perpendicular to the sun, was 98%. The data were taken from measurements for the reference values, for altitude 5° and azimuth 180°. The model was secured to a box one metre high so that the height of the tube's entrance matched the centre of the artificial sun.

The laboratory measurements for the diffuse sky were performed in the sky-dome simulator, where the dome luminance distribution refers to the CIE Standard Overcast Sky, in which the zenith luminance is three times that of the horizon. In such a sky-dome simulator, the radius of the dome was large enough to allow for the models of reasonable size to be tested without causing parallax. The model itself could not be too small and should allow for the details necessary to the light simulation [1]. Therefore, to hold the parallax error under the +−10%, the radius of the simulator should allow for a minimum 10 times the size of the model. As discussed in Lynes and Gilding [11], this method applies only if the model is placed at the centre of the dome.

**Fig. 7:**
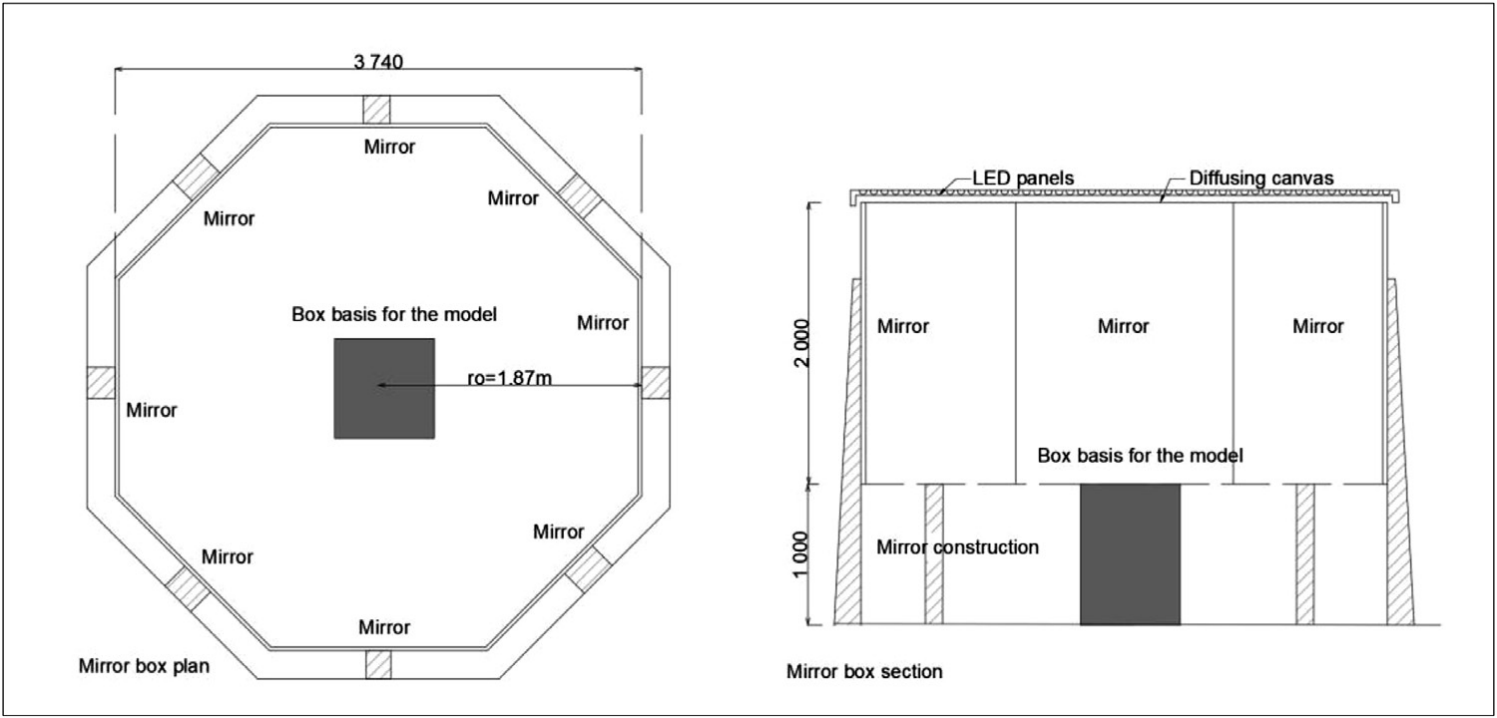
Mirror box for the artificial overcast sky study at NTNU, Faculty of Architecture and Design, in plan (left) and section (right) [18].

In the original study, the test with diffuse light was performed in an artificial sky in the form of a mirror box (Fig. 7). The mirror box was initially developed between 2000 and 2003 with fluorescent tubes and a translucent fabric suspended between the tubes and mirrors. In 2012, the tubes were replaced by LED (RGBW) chips and the fabric by translucent acrylic ceiling plates. The box was octagonal, ensuring more even horizontal light distribution than rectangular mirror boxes with slightly lower luminance in the vertical corners than at the mirror centres. An octagonal box gives users more flexibility in the rotation of the model because it does not matter if the daylight opening in the model is oriented toward a mirror centre [13,14].

Because the height of the tube's entrance is 150 mm, 7.5% of sky height in the mirror box (2000 mm), the parallax error was estimated to be somewhat higher than 10% for low altitude angles (0°–15°). For altitude angles over 15°, the parallax error was lower than 10% [11]. The test model was attached to the table located in the middle of the mirror box. The table height was adjusted to align with the lowest edge of the mirrors. The tube was placed with the opening at the centre of the mirror box. This location was based on the fact that the overcast sky simulated in the artificial sky chamber was rotationally symmetrical. That is, its luminance distribution was not dependent on the azimuth angle.

To obtain reliability of the photometric measurement in a laboratory conditions a certain level of accuracy is necessary to ensure, in all steps. First, it is essential to ensure a stability of supply voltage for the power operated light sources and illuminance meters. Requirements and operating conditions for the light source should be provided, e.g. stabilization and cooling time of the lamps, ambient temperature, and a maximum air-movement speed. To ensure measuring instrument's precision, short-term repeatability tests should be performed prior to each measuring session. Calibrated illuminance meters, with suitable measuring range for the purpose, should be used.

Since such laboratory tests can be long-lasting, but not all physical conditions in the laboratory are perfectly stable (dust), the accuracy of the obtained data and the reliability of the entire measurement should be strengthened by repeatability, by repeating one single measurement, for example, or all of them. . If the measuring method is performed in only one laboratory, a repeatability test can be performed for part of the study, e.g. only for one alternative.

#### Laboratory measurements and calculation of the light transmission efficiency (*η*)

The empirical study was conducted on a scale model of the horizontal light pipe (HLP). The 1:2 scale model of the light pipe was 150 mm in diameter and 1200 mm long. This light pipe was the most common pipe module on the market; a 300 mm diameter pipe also suggested by the manufacturer for use in a single office. The pipe was coated with specular mirror folium with 99% reflectivity (Specular silver film DF2000MA, 3M). The pipe did not have a dome on the entrance or a diffuser on the exit because the original research aimed to study the collection efficiency of the LCP as a custom-made light collector. The assumption was that the LCP could be the “dome’’ or outside enclosure of the pipe. The diffuser was constructed in the shape of a curved reflector, a more efficient light distributor (see [18]).

**Fig. 8:**
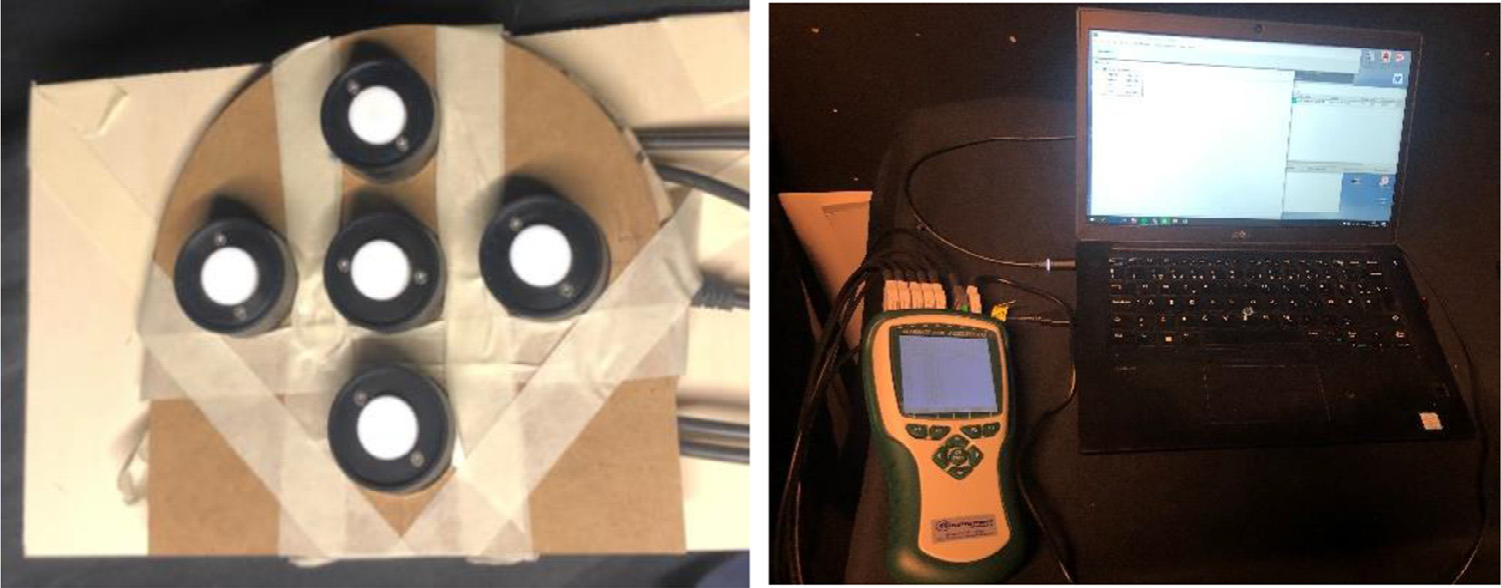
(Left) Measurement instrument using Almemo Ahlborn, with photosensors fixed on a circular plate and placed at the tube's exit; (right) logging of measured data via Ahlborn Almemo logger and Almemo control 6.0 software.

The model was attached to a box one m high so that the height of the tube's entrance matched the centre of the artificial sun or the lowest edge of the mirrors in artificial overcast sky. For the altitude variation measurements (Table 1), the model was tilted by lifting the backside onto a special vertical shelf; azimuthal variation measurements were taken by rotating the box to align it with the angle grid on the floor. Lighting measurements were then taken with five Almemo photosensors arranged in a cross on a circular surface. The results were logged via an Ahlborn logger and recorded using Almemo control software 6.0 (Fig. 8).

**Fig. 9:**
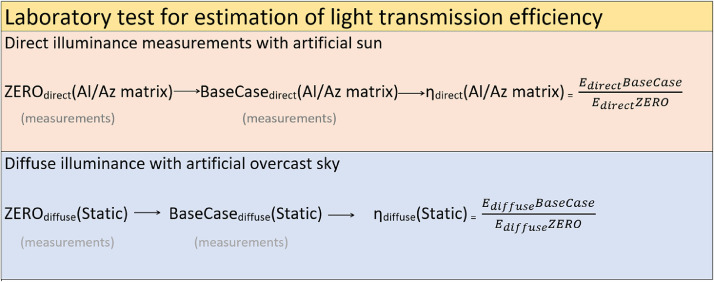
Parametric study method and findings of the light transmission efficiency *η* (for direct and diffuse).

The photometric test for direct light simulation was conducted in all matrix positions, while the diffuse light simulation was conducted for just one fixed position. The measurements were taken for the reference values, called ***ZERO,*** by placing the sensors in front of the tube's entrance, as well as for the test values, called ***BaseCase***, by placing the sensors on the tube's exit (Fig. 9). This nomenclature was used in the original study, the aim of which was to test the efficiency of the LCP samples and BaseCase (where BaseCase presents ***none-LCP*** was then taken as a reference to compare it with). For each measuring position on the matrix, the light transmission efficiency, *η*_direct_, was derived from the ratio of the illuminance (taken by the photosensors) on the tube's exit, *E*_direct_(BaseCase)(Al/Az matrix), and the illuminance measured on the tube's entrance, *E*_direct_(ZERO)(Al/Az matrix) (Eq. (4)). The light transmission efficiency, *η*_diffuse_, was found from the ratio of the illuminance on the tube's exit, *E*_diffuse_(BaseCase)(static), and the illuminance measured on the tube's entrance, *E*_diffuse_(ZERO)(static) (Eq. (5)).

(4) $$\eta_{direct}\left(Al/Az\;matrix\right)=E_{direct}\left(BaseCase\right)÷E_{direct}\left(ZERO\right)$$

(5) $$\eta_{diffuse}\left(static\right)=E_{diffuse}\left(BaseCase\right)÷E_{diffuse}\left(ZERO\right)$$

### Satel-Light database and real daylight accessibility

**Fig. 10:**
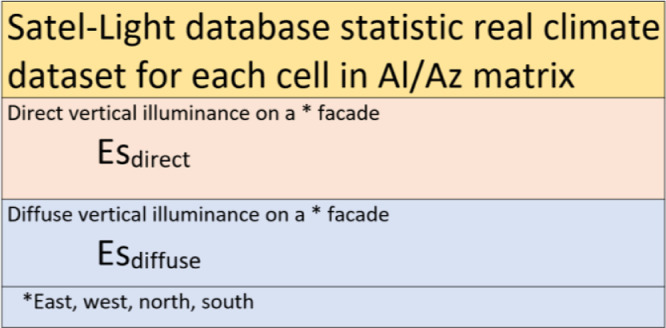
Method for the development of Satel-Light data, *E*s_direct_ and *E*s_diffuse._

The resulting light transmission efficiency **η**_direct_ and **η**_diffuse_ are raw data which, under real conditions, would depend on natural sunlight/skylight conditions at a specific temporal point. In addition to the sun's altitude, the direct and diffuse light intensity depends substantially on the climate at the location and season of the year. For this purpose, statistical data on vertical illuminance for the chosen orientation can be retrieved from the Satel-Light database (Fig. 10). The Satel-Light differentiates between direct and diffuse illuminance values and, in this case, offers the opportunity to estimate daylighting under real conditions. An hourly set provided with the data can be used, together with *solar chart table of changes* in the solar altitude and azimuth angles (for a typical month) to reference the values to the specific matrix cell Al*Az* (Section 2a*"Development of temporal template using a matrix of altitude and azimuth"*). Each matrix cell could be referenced to a specific month and time.

### Development of performance indices using Satel-Light database

**Fig. 11:**
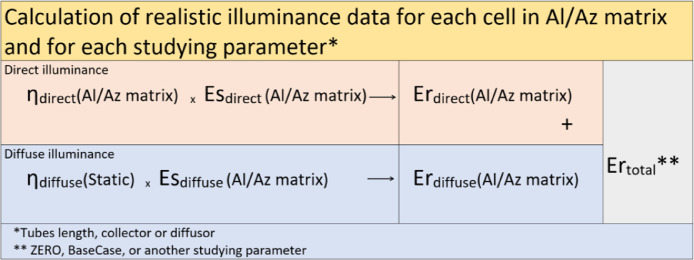
Procedure for developing the real illuminance values for direct and diffuse light and a resulting illuminance.

The values for direct and diffuse illuminances that occur in reality were used to develop indices of real illuminance values that could be realistically expected on the tube exit (Fig. 11). Actual values of the illuminances on the tube's exit, *Er*_direct_ and *Er*_diffuse_, were developed by multiplying the light transmission efficiency, ***η_direct_*** and ***η_diffuse_***, and the illuminance values from the Satel-Light database*, Es*_direct_ and *Es*_diffuse_ (Eqs. (6) and (7)). Final *Er_total_* was a result of the summation of *Er_direct_* and *Er_diffuse_* for any position from the matrix. For each position in the matrix, the *Er*_total_ gives the indices of the real expected illuminance on the tube's exit.

(6) $$Er_{direct}\left(Al/Az\;matrix\right)=\eta_{direct}\left(Al/Az\;matrix\right)\times Es_{direct}\left(Al/Az\;matrix\right)$$

(7) $$Er_{diffuse}\left(Al/Az\;matrix\right)=\eta_{diffuse}\left(Static\right)\times Es_{diffuse}\left(Al/Az\;matrix\right)$$

(8) $$Er_{total}\left(BaseCase\right)=Er_{direct}\left(BaseCase\right)+Er_{diffuse}\left(BaseCase\right)$$

This paper is supplemented with the data from the original study for the *Er_total;_* for the BaseCase, and examples of special collector (see also Section *"Example of an application: an estimate of light transmission efficiency for light deflecting panels as collectors for a horizontal light pipe"*), in form of tilted (T) LCP and rotated (R) LCP (Tables 3, 4 and 5).

### The application of real data via the theoretical office model

To analyse the results of *Er_total_*, a theoretical concept of an imaginative working space was employed. The concept assumed that illuminance in the working area was provided only through the horizontal light pipe. The straight horizontal tube, with an aspect ratio of 8, was assessed in the theoretical model of the office space. The tube was 2.4 m long. Assuming a wall thickness of 30 cm, the tube's exit was 2.1 m from the facade wall, which corresponds to the second working area from the window. The reference illuminance of 300 lux on a reference surface was taken as a threshold for verification, and the necessary threshold illuminance value on the tube's exit was calculated using the inverse-square law. This threshold value was used to verify the *Er*_total_, the real expected illuminance value for each cell from the template, to confirm whether the light pipe in that temporal position supplied room with enough light. For a detailed description of this calculation, please consult the original research paper:Daylight autonomy improvement in buildings at high latitudes using horizontal light pipes and light-deflecting panels, B. Obradovic and B. S. Matusiak, Solar Energy 2020 Vol. 208 Pages 493-514, DOI: https://doi.org/10.1016/j.solener.2020.07.074.

### Example of an application: an estimate of light transmission efficiency for light deflecting panels as collectors for a horizontal light pipe

The proposed customized method is applicable for estimating light transmission efficiency for several parameters in a horizontal light pipe in the same way as the original method suggests. The method can be used to establish the efficiency relationship between different pipe lengths, or the transmission efficiency of a light collector or light diffuser/distributor.

The research studies on horizontal light pipes often draw attention to improvement of the light collection issue. Even in the case of an highly highly reflective pipe, the increased inter-reflection of light rays reduces the output; the longer the pipe (deeper building), the higher the number of reflections. This issue occurs when incident light comes from oblique angles (e.g., morning or evening light from east and west for a south-oriented pipe). Therefore, the original study used light deflection panels, popularly known laser-cut panels (LCP) [4,5], to change the incident angle of incoming unfavourable light rays and direct them in parallel along the pipe's longitudinal (central propagation) axis. In this way, the LCP plays the role of a collector of incoming light.

In the original study, several LCP configurations were used. Some of them were tilted (T), and some were rotated (R). The transmission efficiency *η*_collector_ for a one LCP configuration could be developed, (using steps from Section *"The existing method for calculating the light transmission efficiency of light pipes"*and further) as a ratio of illuminance *E* (LCP configuration) and *E* (BaseCase) for each measuring point from the matrix in Section *"Development of temporal template using a matrix of altitude and azimuth".* The (*η*) of only the LCP configuration as a collector (called T–R in the original study; please consult the original paper for more information on LCP configurations) could be developed separately for both direct and diffuse light, as proposed in Eqs. (9) and (10).

(9) $$\eta collector{\left(T-R\right)}_{direct}\left(Al/Az\;matrix\right)=E_{direct}\left(T-R\right)÷E_{direct}\left(BaseCase\right)$$

(10) $$\eta collector{\left(T-R\right)}_{diffuse}\left(static\right)=E_{diffuse}\left(T-R\right)÷E_{diffuse}\left(BaseCase\right)$$

The resulting *η*_collector_(T–R) for one LCP configuration in all matrix positions is just raw data, and the comparison between the collectors’ performance in this phase can be made just between the single matrix cells. To evaluate the real expected illuminance, the *η*_collector_(T–R) can be further used in steps described in Fig. 10, Fig. 11. In that case, however, it must be multiplied by the *η*_direct_ or *η*_diffuse_ that describe the efficiency of the pipe (described in Section *"Laboratory measurements and calculation of the light transmission efficiency (η)"*).

This method is applicable for any collector the performance efficiency of which depends essentially on incident angles of light rays. In recent decades, research on horizontal light pipes also considered the anidolic collector in addition to the LCP [16]. The shape of the anidolic collector is described by a parabola through edge rays principles [19,22]. The anidolic collector for horizontal light pipes bases its efficiency on a zenithal skylight (Satel-Light data for horizontal diffuse illuminance should be used); however, the curvature of the collector ensures that direct sunlight rays (from lowest to highest altitude) will be captured by the collector as well. The measuring matrix developed for specific altitude angles must, in the case of such collectors that are sensitive to incident angles, be developed with attention to those angles.

## Declaration of Competing Interest

The authors confirm that there are no conflicts of interest.

## Figures and Tables

**Table 3:** BaseCase Er (lux) values.

**Table 4:** LCP T-06-27 Er (lux) values.

**Table 5:** LCP T-05-20 Er (lux) values.

## Appendix

**Er_total_ data-matrix from the original study (LCP as a collector of the horizontal light pipe oriented south and located in Oslo, Norway)**

Er_total_ data-matrix from the original study (LCP as a collector of the horizontal light pipe oriented south and located in Oslo, Norway). The examples of the Ertotal matrix for the BaseCase and LCP configurations are presented in Tables 3, 4 and 5. The colours in the cells correspond to the periods described in Figure 5 (red-summer, orange-late spring or early autumn, yellow-early spring or late spring, blue-winter).
